# Frame loads accuracy assessment of semianalytical multibody dynamic simulation methods of a recreational vehicle

**DOI:** 10.1007/s11044-020-09756-8

**Published:** 2020-07-27

**Authors:** Nicolas Joubert, Maxime Boisvert, Carl Blanchette, Yves St-Amant, Alain Desrochers, Denis Rancourt

**Affiliations:** 1 Department of Mechanical Engineering, Université de Sherbrooke, Sherbrooke, Canada; 2 Department of Mechanical Engineering, Université Laval, Québec, Canada; 3 Centre de Technologies Avancées BRP, Université de Sherbrooke (CTA), 3000 Boulevard de l’Université, Sherbrooke, QC J1K 0A5 Canada

**Keywords:** Load prediction, Semi analytical, Wheel force transducer, Flexible multibody modeling, Sensitivity study, Frame loads

## Abstract

The design of a vehicle frame is largely dependent on the loads applied on the suspension and heavy parts mounting points. These loads can either be estimated through full analytical multibody dynamic simulations, or from semi-analytical simulations in which tire and road sub-models are not included and external vehicle loads, recorded during field testing, are used as inputs to the wheel hubs. Several semi-analytical methods exist, with various modeling architectures, yet, it is unclear how one method over another improves frame loads prediction accuracy.

This study shows that a semi-analytical method that constrains the vehicle frame center of gravity movement along a recorded trajectory, using a control algorithm, leads to an accuracy within 1% for predicting frame loads, when compared to reference loads from a full analytical model. The control algorithm computes six degrees of freedom forces and moments applied at the vehicle center of gravity to closely follow the recorded vehicle trajectory. It is also shown that modeling the flexibility of the suspension arms and controlling wheel hub angular velocity both contribute in improving frame loads accuracy, while an acquisition frequency of 200 Hz appears to be sufficient to capture load dynamics for several maneuvers. Knowledge of these loads helps engineers perform appropriate dimensioning of vehicle structural components therefore ensuring their reliability under various driving conditions.

## Introduction

Knowledge of vehicle frame loads is of interest in the design process, and the sooner the loads are determined, the easier it is to make appropriate changes to a vehicle design. Determination of these loads with a good degree of confidence helps generate more realistic structures in the course of the first frame design iterations [1].

Vehicle frame loads at the suspension and heavy parts mounting points can be determined by using load sensors installed at the frame mounting points (suspension arms bushings, engine mounts, shock absorbers bushings, etc.). This approach may, however, modify the vehicle dynamic response [2], thus providing unreliable measurements. A second evaluation approach often considered to estimate frame loads is the use of wheel force transducers (WFTs) that monitor forces and moments transmitted at the wheel hubs [3–5]. WFTs are widely used for durability analysis, multibody dynamics (MBD) modeling and handling analysis [6–11]. Although these transducers do not directly provide chassis loads, their data can be used in MBD simulations to predict loads transmitted to the frame. Simulations that make use of WFT data are called semi-analytical, or hybrid simulations in different studies [1, 11–13]. Semi-analytical MBD simulations are valuable since they require neither a tire model nor a road profile, which are essential to perform full analytical simulations.

Full analytical simulations are widely used for handling and primary ride simulations. The vehicle dynamic simulation is conducted in a complete virtual environment, using tire models and a mapped road profile. Full analytical simulations have been used to predict loads exerted on a vehicle frame [14, 15].

In practice, the choice of a full analytical simulation over a semi-analytical simulation depends mainly on the desired analysis and available resources. Full analytical simulations provide more flexibility in the simulated maneuvers, but road mapping, tire models and associated tire characterizations are necessary to build the MBD model. Obtaining these data may be quite expensive when considering numerous roads or multiple tire references that might need to be evaluated. Moreover, depending on the design stage at which the MBD simulations are required, the vehicle tires may not be chosen yet. In contrast, semi-analytical simulations depend neither on road mapping nor on tire models as the main inputs to the model come from experimental data recorded at the hub. Only a test vehicle equipped with the necessary sensors is required. Simulations are limited to the specific test runs conducted in the field, and the vehicle dynamic response may be biased by the instrumentation weight itself. Yet, semi-analytical simulations are useful in many cases, for instance, to determine vehicle frame loads at an early design stage, whereas an existing vehicle version or prototype can be instrumented to capture wheel hub loads. One may then assume that wheel loads on the instrumented vehicle are close to those of the future version. Durability analysis can be performed with WFT data over a higher-frequency spectrum than full analytical models since tire models are limited in their frequency range of validity [13].

In the literature, different semi-analytical simulation methods are proposed. These differ in the specific boundary conditions used to constrain the vehicle frame. For instance, Da Cruz et al. [12] use a half-vehicle model with a fixed and rigid vehicle frame; they applied the recorded WFT forces and moments on the hubs. Tebbe et al. [1] described three different methods. The first one consists in prescribing displacements at the vehicle hubs. These are estimated from a double integration of recorded spindle accelerometer data. They show that it can introduce significant drift in steady-state operating conditions and that the method does not control the sprung mass position. In the second method, WFT forces and moments are applied on the hubs of a fully unconstrained vehicle model. However, they show that due to measurement errors, modeling approximations and numerical errors, this approach turns out to be unstable, causing simulation drift or unwanted behavior after only a few seconds from the beginning of the simulation. In the last method, WFT forces and moments are applied at the hubs, and corrective forces and moments are applied at the vehicle center of gravity (CoG) to ensure that it closely follows a prescribed trajectory, both in position and orientation. A proportional-derivative (PD) controller computes the required corrective efforts. Tebbe et al. [1] showed that predicting frame loads with semi-analytical simulations that include control algorithms of chassis trajectory tend to be more accurate, but neither the control algorithm, nor the effective corresponding improvements were detailed in their study.

Hence, the scope of this paper is to compare frame load prediction accuracy of different semi-analytical methods described in the literature [1, 10, 13]. To that end, an accuracy assessment tool was created to evaluate the performance of each method. The tool compares semi-analytical time domain frame loads to those obtained in a full analytical reference simulation. Finally, the influence of three modeling variants (consideration of suspension arms flexibility, hubs angular velocity control algorithm and semi-analytical input data frequency) on frame loads prediction accuracy were assessed using the most accurate semi-analytical method.

## Methodology

**Fig. 1:**
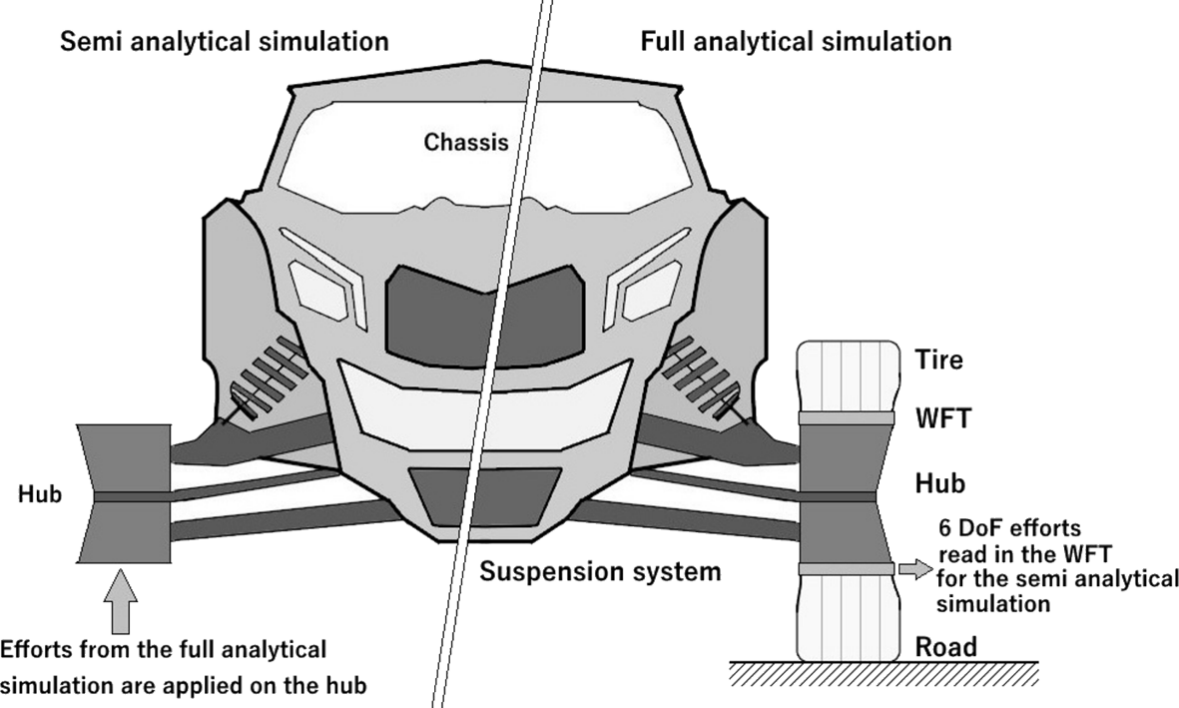
Modeling of wheel assemblies for both full analytical and semi-analytical simulations

The accuracy of four different semi-analytical simulations was assessed in this study. Comparisons with in situ experimental data is not considered in this investigation to eliminate the introduction of measurement errors in the subsequent comparative analysis. Accuracy is defined upon ISO 5725-1 [16], representing both trueness and precision. Accuracy is estimated by comparing, in the time domain, frame load predictions of each semi-analytical method to those obtained from a full analytical vehicle model. The vehicle architecture used to perform the study is described in Sect. 2.1, whereas the full analytical model that is used as a reference is introduced in Sect. 2.2. In full analytical simulations, the tire and a virtual WFT are considered at each wheel, the road profile being taken as a flow source to the model (Fig. 1). In semi-analytical simulations, the tires and the virtual WFTs are not included, and the flow source at the tire changes into an effort source at the hubs.

A detailed description of the four semi-analytical simulation methods is found in Sect. 2.3. They differ in the type and quality of the data required to implement them, as well as in the boundary conditions applied to the vehicle frame, that is:

1. The Grounded Chassis method (GC) that constrains the frame CoG in all six degrees of freedom;
2. The Controlled Chassis around Origin method (CCO) that allows the frame to move in the vicinity of a fixed position through a compliant connection;
3. The Moved Chassis to Recorded Position method (MCRP) that prescribes the movement of the frame via a fixed joint that travels along the recorded vehicle trajectory; and
4. The Controlled Chassis around a Recorded Position method (CCRP) that allows the chassis to move about the recorded vehicle trajectory.

The last two methods require frame trajectory data, both in position and orientation. In this paper, both are obtained from the full analytical simulations.

The comparison tool used to assess the accuracy of each method is detailed in Sect. 2.4. A sensitivity study on three modeling variants driven by the availability and quality of acquisition data is finally described in Sect. 2.5. Indeed, by using the same vehicle model in the reference (full analytical) and the semi-analytical simulations, it becomes possible to isolate the effects of each modeling variant (e.g. boundary conditions, model simplifications or sensor sampling frequency) on the frame load prediction accuracy.

### Vehicle MBD model

**Fig. 2:**
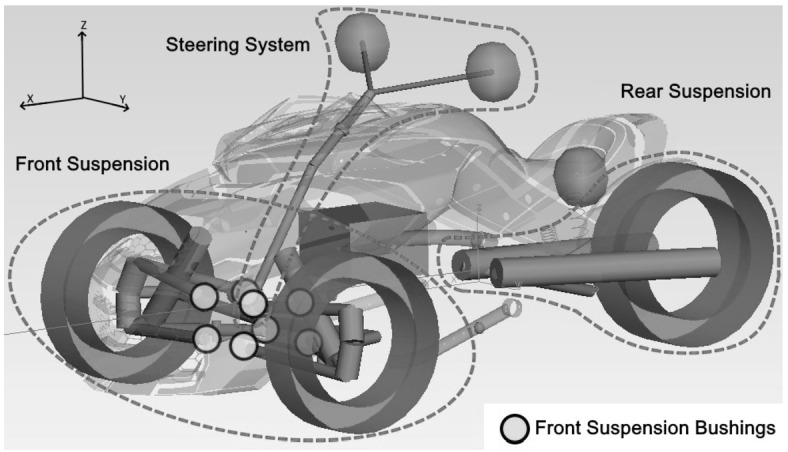
MBD model of the three-wheeled vehicle

The vehicle used in this study is a three-wheeled recreational road vehicle with two front wheels and one rear wheel (Fig. 2). The focus of this study being the development of a comparative methodology to assess load prediction accuracy, rather than the development of an MBD model of a vehicle for its own sake, an extensive description of the vehicle parameters is therefore beyond the scope of this study. Briefly, the steering system consists of a handlebar, a steering column with one universal joint and a pitman arm. The steering system is only active on the front wheels. The front suspension is a double wishbone configuration with an upper and lower A-arm on each side; a helical spring and a shock absorber parallel to the spring are connected to the lower A-arm. Bump and rebound stops nonlinearities are included in the springs force-displacement curves. The front suspension includes front and rear bushings on the A-arms mounting points to the frame, as highlighted in Fig. 2. These bushings are modeled as six degrees of freedom linear spring-damper components. The rear suspension is composed of a single pivot swing arm with a helical spring including bump and rebound stops and a shock absorber. The rear suspension pivot is modeled as a single revolute joint as it is mounted on two coaxial rigid bearings on the actual vehicle. The engine is modeled as a rigid body supported by four distinct engine mounts, without drivetrain elements connecting it to the rear wheel. The driver is modeled as five lumped masses rigidly connected to the seat, handlebar and foot pegs. All model components are modeled as rigid bodies. SimPack^TM^ version 2018x (Dassault Systèmes, Vélizy-Villacoublay, France) was used to build the MBD model. For the full analytical simulations, both front and rear tires are simulated using an MF tire model characterization [15, 17].

### Full analytical simulations

**Fig. 3:**
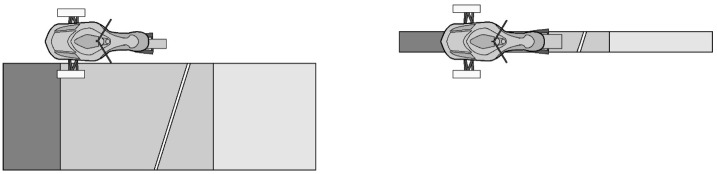
Single wheel ramp maneuvers: (left) ramp under left wheel, (right) ramp under rear wheel

Full analytical simulations were first performed to compute reference signals that are further used to assess the performance of the semi-analytical simulations. The full analytical simulations generated both reference WFT and frame loads signals. WFT signals were further used as inputs to the semi-analytical simulations, whereas frame loads signals were used to assess the semi-analytical load prediction accuracy. Four specific maneuvers were investigated in this paper, each of them creating high dynamic loads along specific axes:

1. **Slalom**: the vehicle is launched at approximately 30 km/h, and the steering is excited with a sinusoidal prescribed motion of the steering column joint. The sine amplitude is sufficient to lift the inside front tire. This maneuver highly excites both yaw and roll dynamics.
2. **Single wheel ramp**: the vehicle is launched at approximately 30 km/h on a flat surface with a downhill ramp located on the wheel trajectory. This maneuver, applied to each side of the vehicle and on the rear wheel, is illustrated in Fig. 3, and the ramp profile is shown in Fig. 4. The maneuver excites both roll and pitch dynamics. In this maneuver, the steering column joint is locked in place at 0° orientation.

**Fig. 4:**
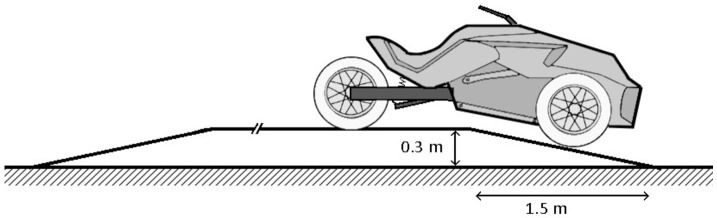
Steep downhill ramp
3. **Steep downhill ramp**: The vehicle is launched at approximately 30 km/h on a flat surface. Then it moves forward on an uphill smooth ramp, wider than the vehicle, followed by a long flat surface, then a steep downhill ramp, and a final flat surface, as shown in Fig. 4. The front wheels, followed by the rear wheel, loose ground contact during the maneuver, which causes high suspension displacement, exciting pitch dynamics. In this maneuver, the steering column joint is locked in place at $0^{\circ}$ orientation.

4. **Hard braking**: The vehicle was launched at 110 km/h on a flat surface, and a braking torque was applied on each wheel to stop the vehicle. A control algorithm computed the torques needed on the wheels to generate 5% longitudinal slip on the tires. This maneuver highly excited pitch dynamics and caused high longitudinal decelerations. Again, in this maneuver, the steering column joint is maintained at 0° orientation.

**Fig. 5:**
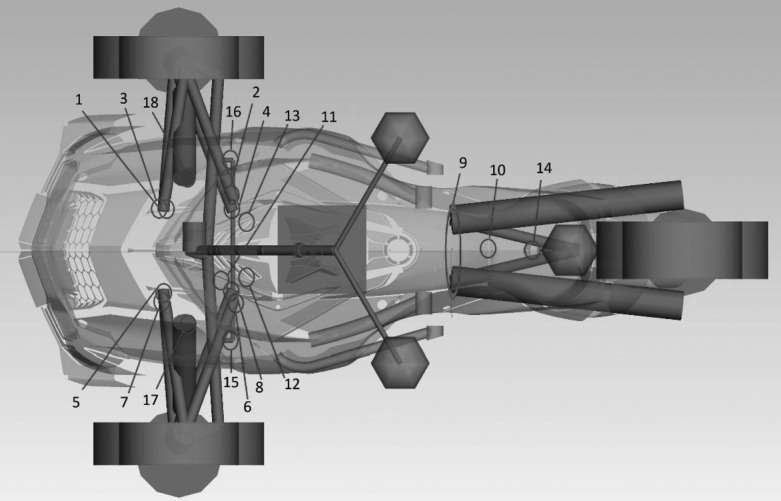
Suspension and heavy parts 18 mounting points
- Wheel angular velocities;
- Frame CoG position and orientation;
- Shock absorbers length;
- Steering orientation; and
- Three-dimensional forces and moments transmitted to the hubs;

The following signals are extracted from the full analytical simulations:

- Frame loads transmitted by the suspension and heavy parts to their 18 mounting points (Fig. 5);

Extraction of the forces and moments transmitted to each hub is achieved, in the full analytical simulations, through a virtual WFT by modeling the wheel assembly with two different bodies: the hub, the rim and tire, connected with a fixed joint. Loads in the joint are those that a WFT would measure, as this modeling reproduces the construction of a real WFT. The WFT does not read the inertial effects created by the hub and the spindle, but those of the tire and rim along with the efforts transmitted by the tire to the hub. The loads are expressed in the knuckle local coordinate system.

### Semi-analytical simulations

Among the four semi-analytical methods studied in this paper, the first two require only forces and moments at the hub, whereas the other two also need vehicle frame trajectory data. In this study, a full analytical model is used to generate them. In the semi-analytical simulations, WFT signals computed in the full analytical simulation are applied to the hubs. The steering orientation is prescribed, following the steering orientation recorded in the full analytical reference simulations.

In all semi-analytical methods, efforts are applied to the hubs, which are unconstrained along their rotational axis. The virtual WFT signals generated by the full analytical simulation provide the brake torque transmitted between the knuckle and the hub, and the inertial torques to accelerate the hub. Since the hub is allowed to rotate freely and no brakes are modeled, the resulting hub axial torque may accelerate hub rotation up to a point where gyroscopic forces create non-representative loads on the simulation, and significantly biases the simulation. This issue is mitigated by including a viscous resistance in the revolute joint between the hub and the knuckle.

#### Methods for which no frame trajectory data is needed

In the Grounded Chassis method (GC), the vehicle frame is held at a fixed point in the global fixed reference frame via a fixed joint. The vehicle frame cannot move or rotate about the joint, and therefore only the suspension movement is simulated since the chassis is modeled as a grounded rigid body.

In the Controlled Chassis around Origin method (CCO), the vehicle frame is unconstrained, but a proportional-derivative control algorithm computes the required forces and moments to apply on the frame CoG to maintain it close to its initial position and orientation. The control algorithm acts as a six degrees of freedom linear spring-damper connecting the CoG to a fixed point in the global fixed reference frame. This method partially accounts for inertial effects as limited motions of the chassis are allowed. However, biases should be expected in inertial force amplitudes and directions or in vehicle steady accelerations.

#### Methods that need frame trajectory data

The second set of semi-analytical simulations requires prior knowledge of the frame position and orientation over the whole simulation time period. Such data are extracted from the full analytical model and then used as a control reference trajectory in the semi-analytic models, such that no measurement noise issue needs to be addressed in the performance analysis.

In the Moved Chassis to a Recorded Position method (MCRP), the frame CoG is rigidly attached via a fixed joint to a moved marker that follows the frame’s CoG position and orientation trajectory, extracted from the full analytical simulation.

Finally, the Controlled Chassis around Recorded Position method (CCRP) is very similar to the CCO method. The main difference arises from the fact that forces acting at the CoG are computed by a proportional-derivative controller that controls the frame CoG to the moved marker position and orientation extracted from the full analytical simulation. The controller acts independently along each axis.

### Accuracy assessment of frame load predictions

Accuracy assessment of the four semi-analytical methods is achieved by comparing three-dimensional forces and moments time varying curves at the suspension and heavy parts mounting points on the vehicle frame, to those obtained from the full analytical model for four different maneuvers. A total of 18 suspension and heavy parts mounting points were considered in the accuracy assessment study. These points are located in Fig. 5 and listed in Table 1.

**Table 1:** List of all suspension and heavy parts mounting points

Point #	Sub system	Identification name	Description
1	Front suspension	AarmDrSupAv2Chas	Right upper A-arm, front bushing
2		AarmDrSupAr2Chas	Right upper A-arm, rear bushing
3		AarmDrInfAv2Chas	Right lower A-arm, front bushing
4		AarmDrInfAr2Chas	Right lower A-arm, rear bushing
5		AarmGaSupAv2Chas	Left upper A-arm, front bushing
6		AarmGaSupAr2Chas	Left upper A-arm, rear bushing
7		AarmGaInfAv2Chas	Left lower A-arm, front bushing
8		AarmGaInfAr2Chas	Left lower A-arm, rear bushing
9	Rear suspension	SArmPvt2Chas	Swing arm pivot
10	Engine	MotBushingCentre	Engine center bushing
11		MotRodFrameSide	Engine rod
12		MotTampG	Engine left bushing
13		MotTampD	Engine right bushing
14	Rear suspension	SusAr2Chas	Rear suspension shock absorber bushing
15	Front suspension	BStabGa2Chas	Left sway bar bushing
16		BStabDr2Chas	Right sway bar bushing
17		SusAvG2Chas	Front left suspension shock absorber bushing
18		SusAvD2Chas	Front right suspension shock absorber bushing

Assuming six efforts at each mounting point leads to a total of 108 load signals to be compared in each simulation. To quickly evaluate frame load accuracy, an error map approach was established. The error value is computed as follows.

Let $e$($t$) be the instantaneous error between a semi-analytical model signal prediction $S$($t$) and a full analytical signal reference $F$($t$), defined as

1 $$ e ( t )= F ( t )- S ( t ). $$

One can define a root mean square error and use it to define the following error ratio:

2 $$ \frac{\mathit{RMS} \left ( e ( t ) \right )}{\mathit{RMS} \left ( F ( t ) \right )}. $$

This ratio can be viewed as a normalized error because it considers the energy level of the signal $F$($t$). However, for very small forces or moments, the relative error can become very large because of the low level of energy in the signal $F$($t$). Hence, a modified normalized error is proposed as

3 $$ R= \frac{\mathit{RMS} \left ( g \left ( t \right ). e ( t ) \right )}{\mathit{RMS} \left ( F ( t ) \right )}, $$

where $g$($t$) is defined, for the forces, as

4 $$ g(t)=\min \left ( 1, \frac{\left \vert F(t) \right \vert + \left \vert S(t) \right \vert }{500} \right ) $$

and, for the torques, as

5 $$ g(t)=\min \left ( 1, \frac{\left \vert F(t) \right \vert + \left \vert S(t) \right \vert }{5} \right ). $$

Hence, the error is reduced when the reference signal and the semi-analytical signals are low. A reference threshold of 500 N was arbitrarily chosen for the force magnitude and 5 Nm for the torques. In practice, efforts below those values have negligible impact on the frame design.

**Fig. 6:**
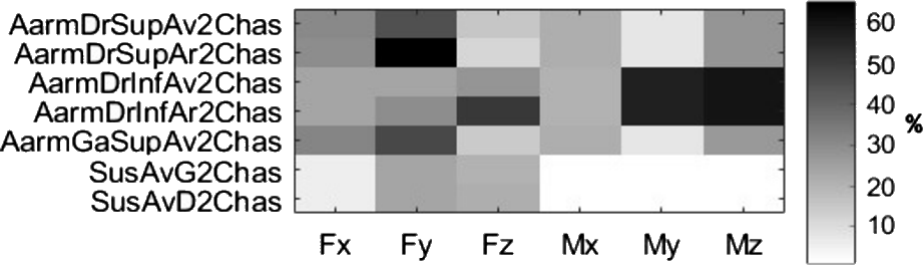
Illustration of the error map along 6 DOF for seven different hard points with errors displayed in %. Each column represents an effort along one of the 6 DOF, whereas each row corresponds to a different mounting point

At each of the 18 mounting points, a modified normalized error was thus calculated along each DOF. This again led to 108 error level values to be examined. These error values can be viewed at a single glance by constructing an error map using a matrix of 18 rows (mounting points) by 6 columns (efforts) error values, where the gray cell shading darkens with the modified normalized error magnitude. Figure 6 shows an arbitrary example of seven mounting points for illustrative purposes.

Gray shadings make it difficult to quantify frame load accuracy. Numbers being easier to compare than a color scale, the comparison tool also included the mean, the maximum and the standard deviation of all 108 errors compiled in the error maps. Those three values are presented in tables that allow a quick evaluation and numerical comparison of the accuracy of each method, whereas the error maps allow for a more global visualization of the results.

### Sensitivity to modeling variants

In practice, field test data may be obtained when the vehicle is not entirely designed, which leads to MBD models of reduced quality. For instance, when conducting preliminary studies, the suspension arms geometry may not be final, and as their flexible representation relies on finite element analysis, they might be modeled, as a first approximation, as rigid bodies. Three simulation variants were considered in this study: the sampling frequency of the instrumentation unit onboard the test vehicle, the rigid body versus flexible body modeling of the suspension arms, and finally, the specific method used to control the angular velocity of each hub.

#### Effect of sampling rate

The sampling rate of a data acquisition system is normally selected based on several parameters such as the number of channels to be recorded, the available bandwidth of the data acquisition system, the memory space available, and so on. While WFTs are able to acquire loads at frequencies up to 2000 Hz, 200 Hz is a common sampling frequency for the inertial measurement units (IMU) and CAN buses used on vehicles. Hence, the sampling rate of the WFT must often be reduced to 200 Hz, and this may have an impact on the prediction accuracy. To measure the effect of a 200 Hz sampling frequency instead of 2000 Hz on frame load prediction accuracy, two semi-analytical simulations were run, with input signals sampled at either 200 or 2000 Hz for the steep ramp maneuver. This maneuver was chosen as it produces high dynamic loads on the vehicle when its wheels contact the surface following a lift off. Frame loads results were then compared to full analytical reference data sampled at 2000 Hz.

#### Influence of flexible body modeling

Although suspension arms are actually flexible bodies, they may not be represented as such in MBD simulations, thereby influencing frame load predictions. The impact of A-arms and rear swing arm flexibilities was therefore studied by comparing load predictions with rigid body arm models versus flexible body models. This was achieved using a Craig–Bampton modal reduction [18, 19] performed on OptiStruct^TM^ (Altair Engineering, Troy, MI, USA), computing modes up to 5000 Hz. Comparisons were made for the steep ramp maneuver and two different vehicle model configurations: one with the four front A-arms represented as flexible elements and one with a flexible rear swing arm. For each case, a flexible full analytical simulation generated reference load data, which were then used as inputs for two semi-analytical simulations featuring rigid and flexible arms, respectively.

#### Effect of hub angular velocity

In all semi-analytical simulations, hub angular velocities could drift and reach very high values if dissipative elements are not incorporated in the hub model. These high angular velocities can create non-representative loads on the suspension components. In Sect. 2.3, it is mentioned that a viscous friction element is used to limit the angular velocity of the hubs at their revolute joint on the hub. In such case, there is no need for recording wheel speeds in the full analytical simulation. Yet, in practice, all WFTs have an accurate encoder that reads the wheel orientation and speed. Hence, in semi-analytical simulations, it becomes possible to use a PD controller instead of a friction element to apply a torque between the knuckle and the hubs and ensure that the hub speed matches the wheel speed signal from the full analytical simulation. This control element reproduces the brake torque applied between the hub and the knuckle. To evaluate the accuracy of both approaches, two semi-analytical simulations were compared to the same full analytical reference for the hard-braking maneuver. In the first simulation, the hubs angular velocities are controlled, whereas, in the other, the hubs angular velocities are limited by a friction element.

## Results

In Sect. 3.1, results for the accuracy assessment of the four semi-analytical methods are given for a steep ramp maneuver, as it is the maneuver that produces the highest loads on the mounting points. Then the accuracy of the best semi-analytical method (CCRP) is presented for the four maneuvers described in the methodology section. Finally, in Sect. 3.2, the effects of the three modeling variants on the prediction accuracy are presented based on the CCRP method.

### Accuracy of the four semi-analytical methods

**Fig. 7:**
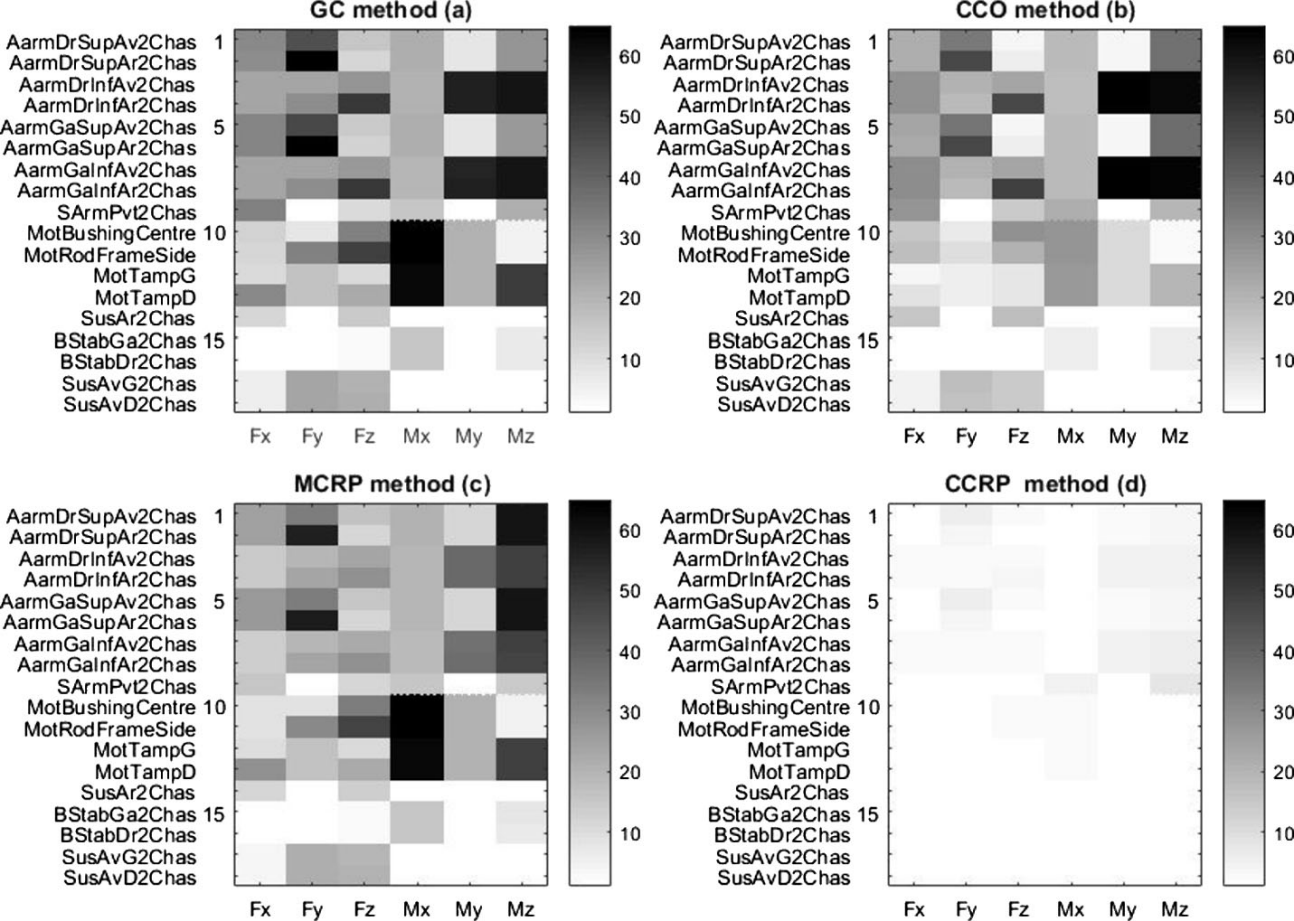
Error maps in % for (**a**) GC, (**b**) CCO, (**c**) MCRP and (**d**) CCRP methods, in the steep ramp maneuver, for the 18 different mounting points

Figure 7 illustrates the error map obtained from the four semi-analytical methods for the steep ramp maneuver, and Table 2 summarizes the corresponding error level statistics.

**Table 2:** Frame load prediction errors statistics for the four semi-analytical methods, for the steep downhill ramp maneuver

Method	Mean error [%]	Max error [%]	Standard deviation [%]
GC	24.3	77.0	19.3
CCO	19.1	67.9	17.9
MCRP	22.0	63.2	17.4
CCRP	1.21	6.43	1.27

The GC method provides a mean error of 24.3%. The main error contributors are the loads originating from the engine mounts, as seen on rows 10 to 13. These loads are not accurate because the frame is grounded, such that inertial effects related to engine accelerations relative to the vehicle frame are not considered.

The CCO method computes and applies the required forces at the CoG to keep the frame close to its original position. Additionally, the inertial effects on the engine are more accurately represented, as can be seen in lines 10 to 13. The results from the suspension loads are somewhat better, but there are still significant prediction errors.

In the CCO and CCRP methods, the control algorithms are simple PD controllers, and their parameters are chosen through manual tuning to minimize frame loads prediction errors. This resulted in a low stiffness and high damping controller for the CCO controller (natural frequency of less than 5 Hz) and a higher stiffness and lower damping for the CCRP controller (natural frequency near 15 Hz).

The CCRP method avoids the issues found in the MCRP method, as the movement of the body results from the loads exerted on it, rather than being directly prescribed by the fixed joint. It provides the best results when comparing the time signals, with a mean error of 1.21 % and a maximum error 10 times inferior to the other methods.

**Fig. 8:**
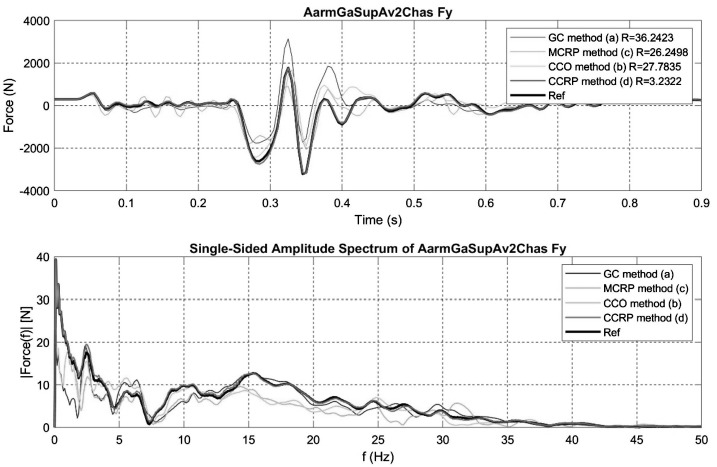
Y axis force on the front bushing of the upper left control arm; comparison of the time and frequency signals of the four different semi-analytical methods for the steep downhill ramp maneuver

To better illustrate the goodness of fit, Fig. 8 illustrates the time and frequency signals of the left, A-arm suspension mounting point (i.e. data corresponding to the fifth row, second column of the error map illustrated in Fig. 7).

**Fig. 9:**
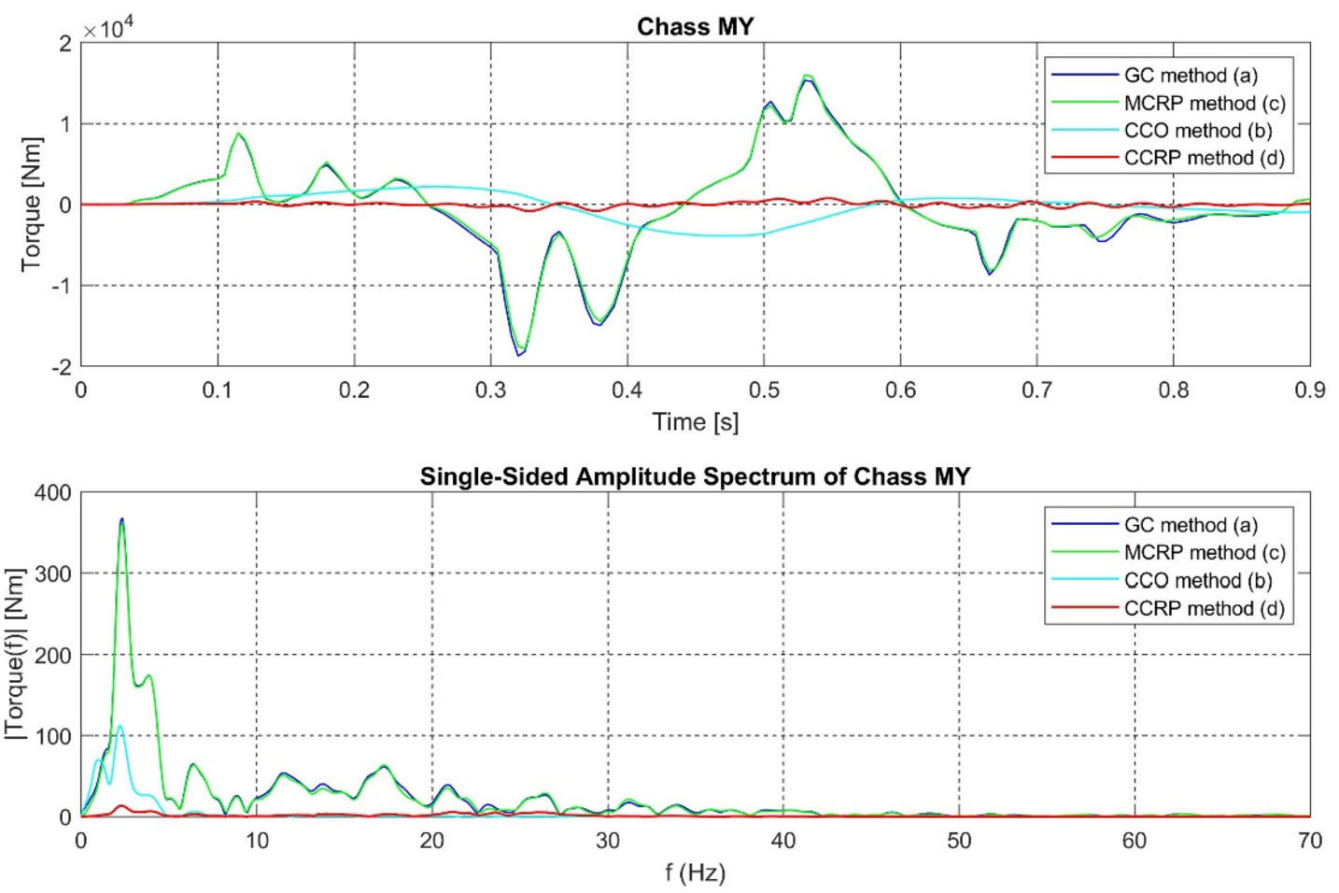
Time and frequency signals for the Y axis torque acting at the vehicle CoG for the four different semi-analytical methods in the steep downhill ramp maneuver

**Fig. 10:**
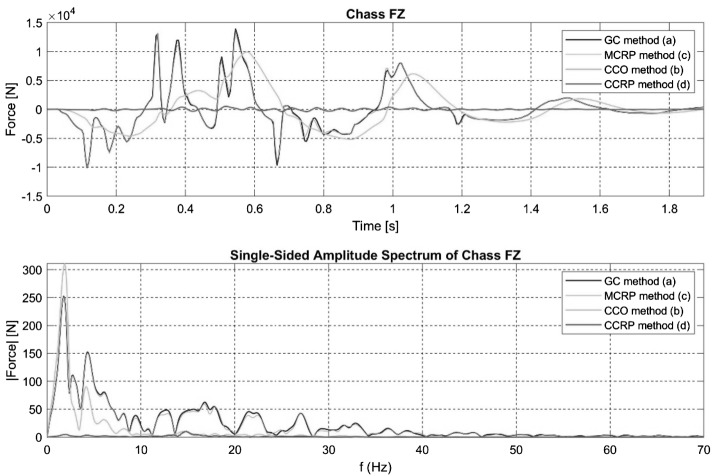
Time and frequency signals for the Z axis force acting at the vehicle CoG in the steep downhill ramp maneuver for the four different semi-analytical methods

Figures 9 and 10 show the time and frequency signals for the My and Fz efforts acting on the vehicle CoG. For the CCRP and CCO methods, these efforts are those produced by the controller. For the CG and MCRP methods, they are reactions due to grounding or trajectory imposed on the CoG. Minimal efforts are therefore observed for the CCRP method as only corrective efforts are applied to the CoG, closely fitting the vehicle frame trajectory to the full analytical generated CoG trajectory. Reaction efforts at the CoG are much larger for both the GC and MCRP methods.

**Fig. 11:**
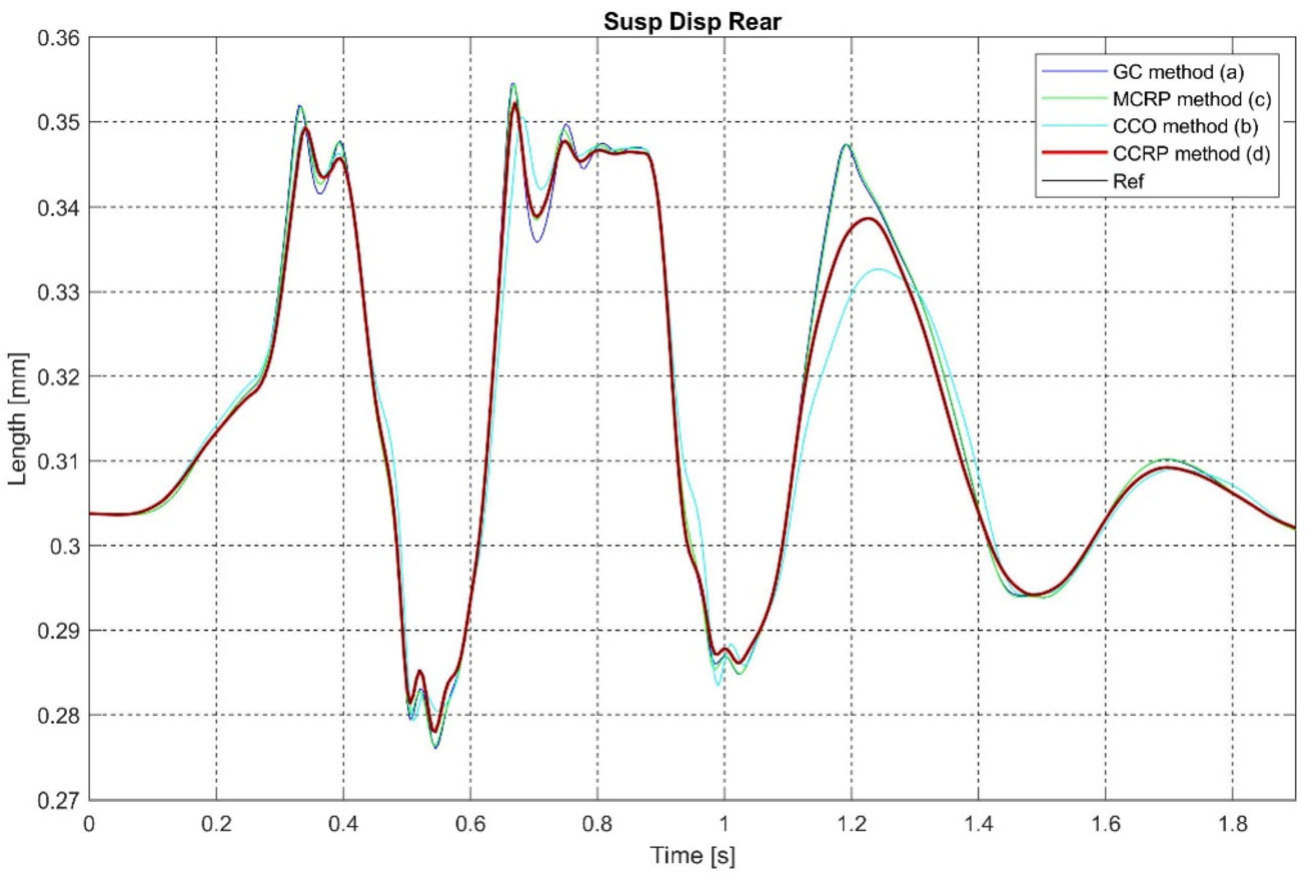
Time signals of rear shock absorber length in the steep downhill ramp maneuver for the four semi-analytical methods

**Fig. 12:**
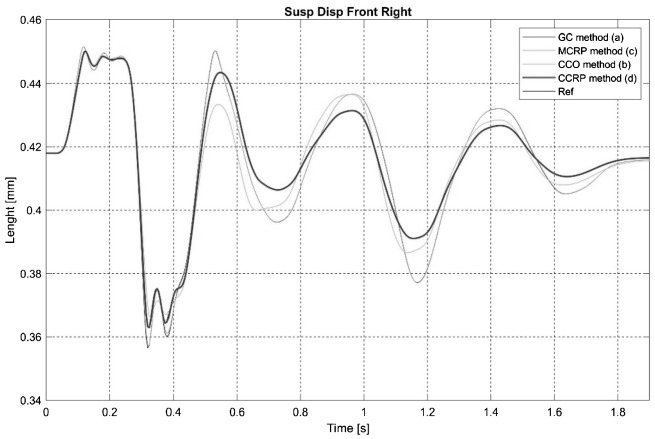
Time signals of the front right shock absorber length in the steep downhill ramp maneuver for the four semi-analytical methods

Figures 11 and 12 compare time signals of the rear and front shock absorber lengths for the steep downhill ramp maneuver for the four semi-analytical methods. Whereas all semi-analytical methods are able to reproduce the signal shape, the CCRP method exhibits the best accuracy overall. In a real-world scenario, the suspension displacement signals are not appropriate to discriminate between semi-analytical methods or to correlate with a model, because the differences observed between the semi-analytical methods are within the accuracy span of common suspension travel sensors and measurement noise threshold.

### Sensitivity of the semi-analytical method to three modeling variants

This section investigates the sensitivity of the CCRP method to three modeling variants that can be caused by the availability or quality of modeling data, namely: sampling rate of the input signals, A-arms and swing arm flexible bodies modeling, and hubs angular velocity control strategy.

#### Influence of input loads sampling frequencies

Fig. 14 and Table 4 compare the results obtained when using the same input signals in the CCRP method on the steep downhill ramp maneuver when sampled at 200 Hz instead of 2000 Hz. The 200 Hz sampling rate is often chosen in commercial DAQ systems used to acquire vehicle data. With this reduced sampling rate, the mean error is increased by a factor of 5, but it is still less than 1%.

#### Influence of flexible suspension arms

Suspension arms were so far considered as rigid bodies. In practice, some vehicle components have flexibilities that could impact forces acting on the frame. Evaluation of this impact was investigated by considering the flexibility of the suspension A-arms and swing arms in the semi-analytical models. When comparing full analytical and semi-analytical simulation results, it was found that semi-analytical simulations predicted mounting point loads with reduced errors, as shown in the left portion of Figs. 15 and 16.

**Fig. 13:**
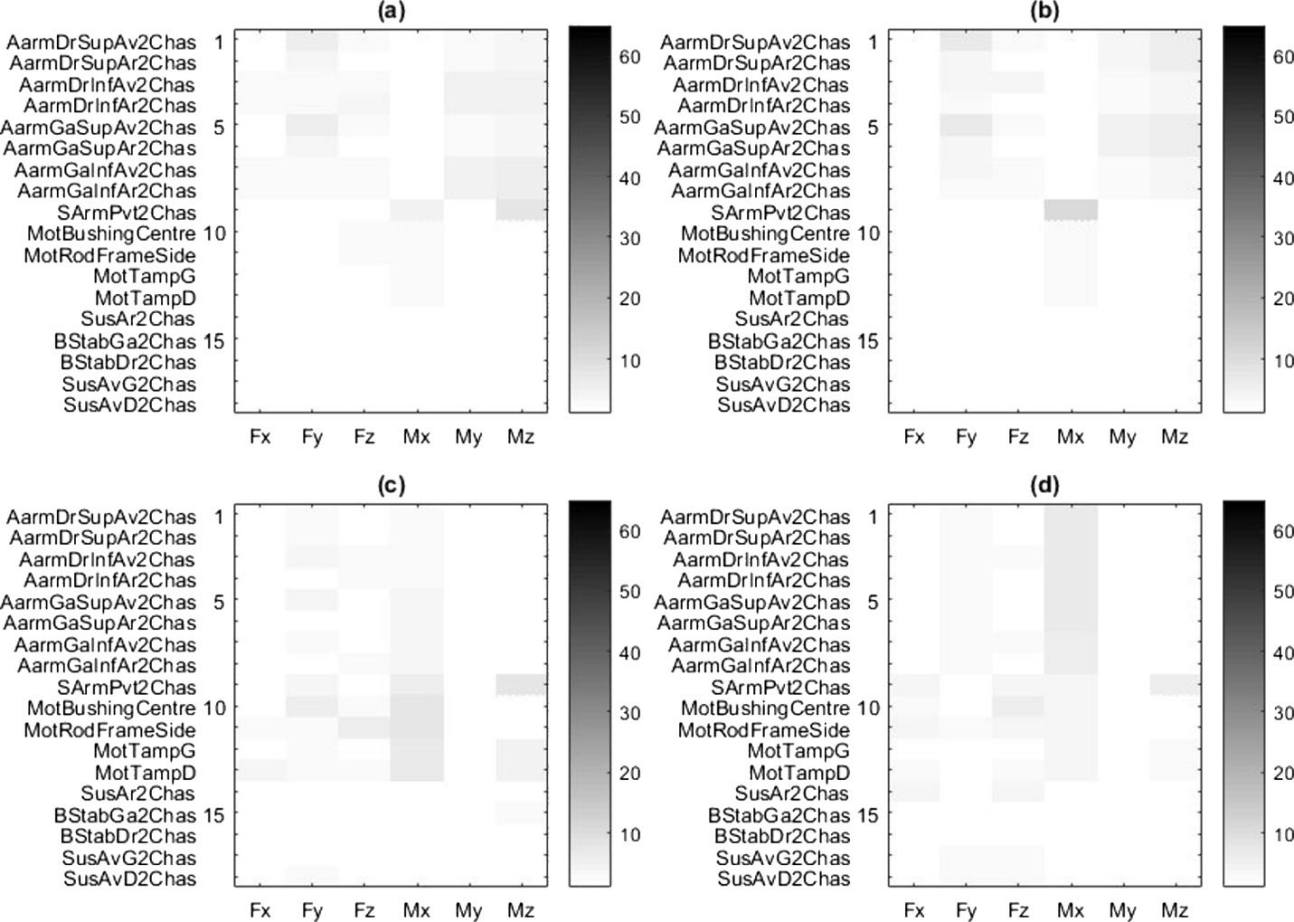
Error level, in %, CCRP method on (**a**) the steep ramp, (**b**) the slalom, (**c**) the right and (**d**) rear wheel ramp maneuvers

The CCRP method was also found to be the most appropriate for the three other maneuvers, whose results are presented in Fig. 13 and listed in Table 3, for reference purposes.

**Fig. 14:**
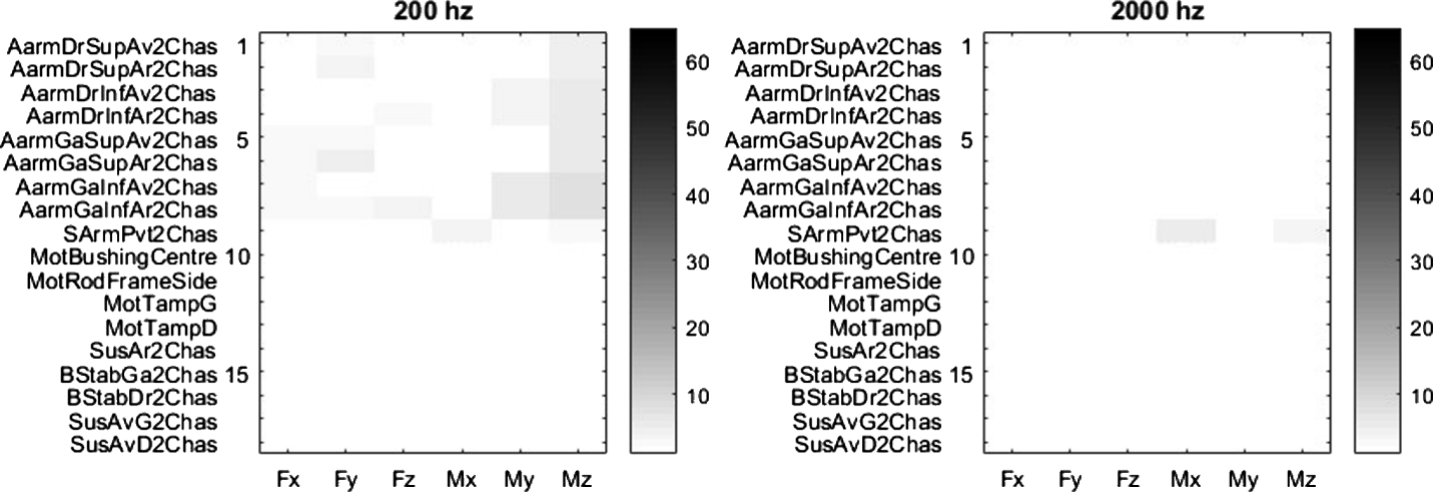
Error levels, in %, for 200 Hz (left) and 2000 Hz (right) input signal sampling frequencies with the CCRP method

**Table 3:** Results summary of the CCRP method in the four reference maneuvers

Maneuver	Mean error [%]	Max error [%]	Standard deviation [%]
Steep downhill ramp	1.2	6.4	1.3
Slalom	1.2	9.3	1.6
Ramp under rear wheel	1.3	5.6	1.5
Ramp under front left wheel	1.2	6.6	1.5

**Table 4:** Results summary for both 200 Hz and 2000 Hz input signal sampling frequencies

Frequency [Hz]	Mean error [%]	Max error [%]	Standard dev. [%]
200	1.0	6.3	1.4
2000	0.2	2.2	0.3

**Fig. 15:**
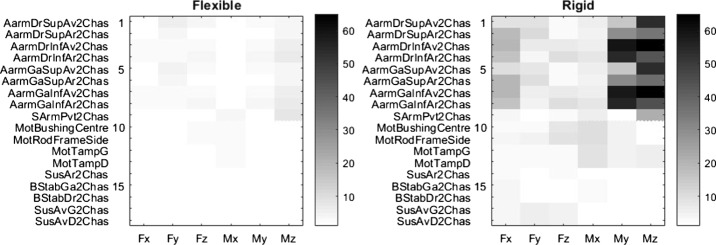
Error levels, in %, with (left) and without (right) flexible A-arms on the steep ramp maneuver with the CCRP method

When using rigid A-Arms in the semi-analytical simulation, the error levels of the mounting points load prediction, displayed in the right portion of Fig. 15, are significantly higher. Table 5 summarizes the results.

**Table 5:** Results summary with and without flexible A-arms on the steep ramp maneuver

Body type	Mean error [%]	Max error [%]	Standard dev. [%]
Rigid A-arms	11.5	88.1	18.0
Flexible A-arms	1.2	6.5	1.3

The errors are mainly associated with moments around the Y- and Z-axes at the A-arms mounting points. Indeed, simulations with flexible arms take into account the tube bending of the A-arms, which does transfer some X-axis force components into the Y- and Z-axes moments at the A-arms mounting points.

**Fig. 16:**
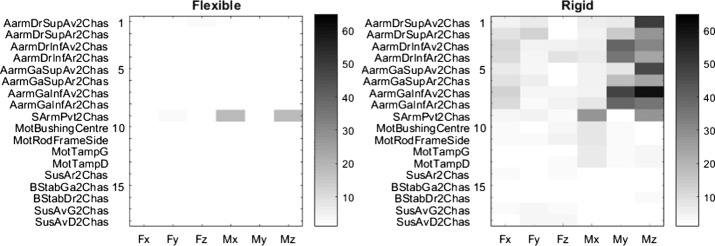
Error level, in %, with a flexible (left) vs. rigid (right) swing arm on the steep ramp maneuver with the CCRP method

The same procedure was repeated to evaluate the impact of modeling a flexible swing arm in the rear suspension. Results are presented in Fig. 16, and Table 6 summarizes the results.

**Table 6:** Results summary with and without a flexible swing arm on the steep ramp maneuver with the CCRP method

Body type	Mean error [%]	Max error [%]	Standard dev. [%]
Rigid swing arm	7.8	59.6	12.5
Flexible swing arm	0.5	17.3	2.2

**Fig. 17:**
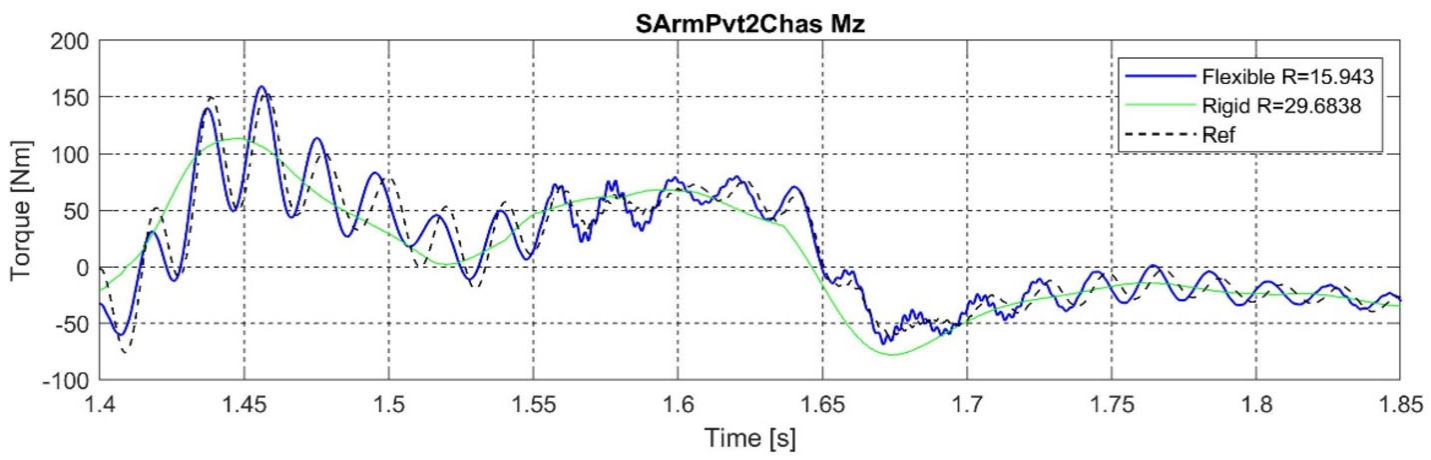
Z-axis torque on the swing arm pivot for the steep ramp maneuver

Results are similar to those with flexible A-arms, except for the Mx and Mz efforts in the semi-analytical simulation with the flexible swing arm. Results illustrated in Fig. 17 compare the Mz signals for the full analytical simulation, the semi-analytical method with the flexible swing arm and the semi-analytical method with the rigid swing arm. In the latter case, we can observe a phase offset between the reference loads and those predicted with the semi-analytical simulation with the flexible swing arm.

This phase offset is clearly visible at a frequency close to the first oscillation mode of the swing arm, which happens to be around 51 Hz. Moreover, the modeling of the swing arm pivot itself increases the induced torques; indeed, it is modeled as a single revolute joint located in the middle of the two bearings that are present on the vehicle. This configuration creates high torques on the single revolute joint, instead of distributing forces on the two opposite bearings.

**Fig. 18:**
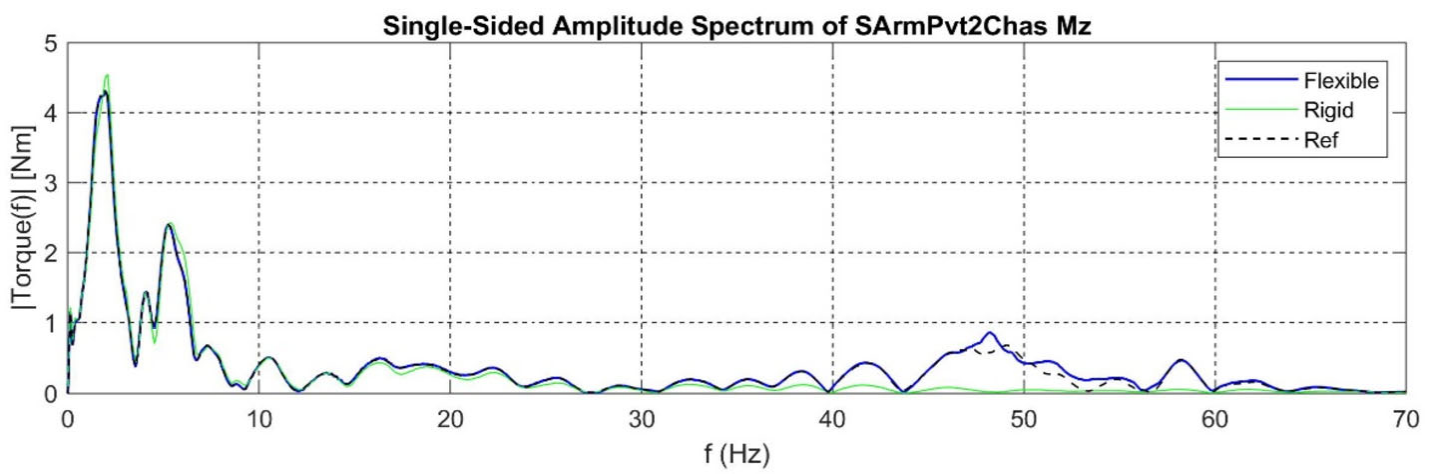
Amplitude spectrum of the Z-axis torque on the swing arm pivot for the steep ramp maneuver

Despite errors due to the phase offset of the two signals, the mean error on all mounting points is as low as 0.5% when considering the flexibility of the swing arm. Moreover, the amplitude spectrum in Fig. 18 shows that the discrepancies between the two amplitude spectrums are not significant, with only a slight error near 50 Hz.

#### Influence of hub angular speed control strategy

**Fig. 19:**
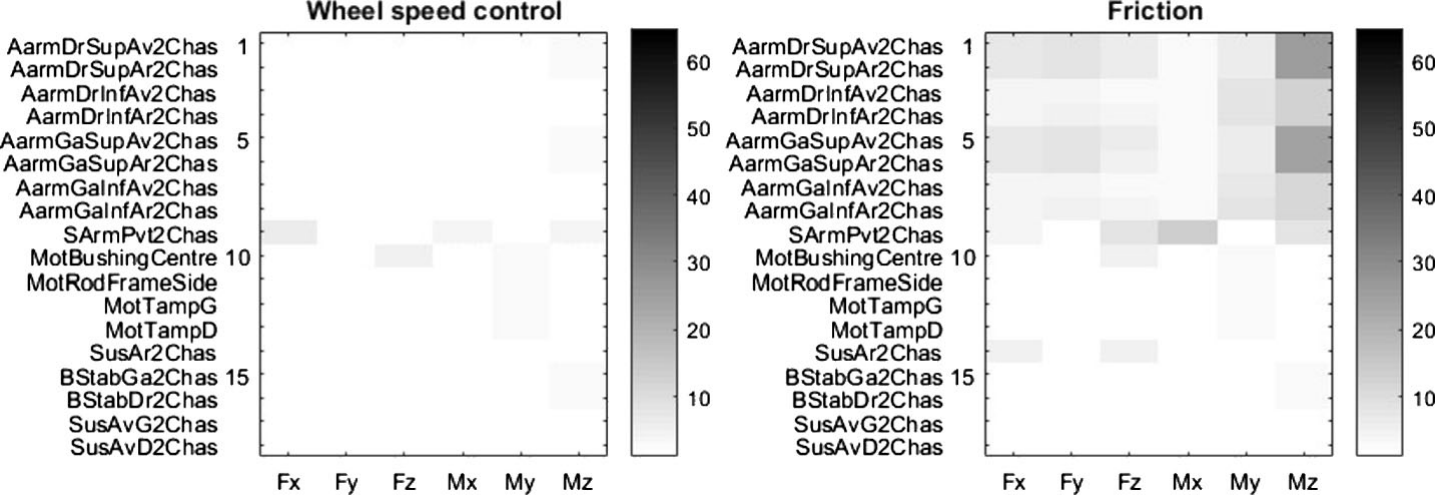
Error level, in %, with hub speed control (left) and hub friction (right) on the hard-braking maneuver with the CCRP method

Results when comparing the full analytical reference with the semi-analytical simulation with controlled hub speed on the hard-braking maneuver are presented in Fig. 19. When comparing results of the semi-analytical simulation with viscous friction elements to the simulation using a control algorithm, the error level increases. Indeed, when a friction torque is applied on the hub of the vehicle, the braking torques are not well represented. Table 7 summarizes the prediction accuracy improvements when changing the hub speed control strategy.

**Table 7:** Results summary with hub speed control on the hard-braking maneuver

Type	Mean error [%]	Max error [%]	Standard dev. [%]
Friction	3.4	22.3	4.7
Control	0.4	4.1	0.7

As noted, the accuracy reduction is 3% on the mean error when the hub speed control is not implemented and viscous damping is used instead. Moreover, the maximum error is reduced by more than 18%. Since the controller does not require more instrumentation on the vehicle than the WFT, it is an easy approach for improving load prediction accuracy, as it models the torques applied by the brakes between the hub and the knuckle

## Discussion and conclusion

Four semi-analytical methods were evaluated on their ability to predict frame loads at several interface points by using an error map evaluation approach based on time domain data. Results show that the CCRP method provides the best results, as the frame is partly unconstrained, and a control element applies forces on it to prevent drifts and instabilities. Inertial effects are well represented in transient accelerations, and vehicle orientation as well as gravitational forces and force orientation applied on the hubs are correctly represented. When compared to full analytical reference simulation data, the CCRP method yields accurate frame load predictions. The mean error is less than 2% for the 18 mounting points evaluated. In contrast, if no trajectory data is available, then the CCO method predicts frame loads with less than 20% error on average.

Model variants were investigated to figure out the impact of practical issues in implementing the semi-analytical model. For instance, the investigation showed that 200 Hz simulations would be sufficient to represent the dynamic behavior of the vehicle. Reducing the sampling frequency from 2000 Hz to 200 Hz increases the maximum error by 4% while decreasing the average performance by only 0.8%, thus showing a marginal improvement for faster sampling rates for the specific tested maneuvers. However, some road maneuvers or obstacles may induce higher frequency excitations that require faster sampling rates.

Results also showed that the addition of flexible components improved the load prediction accuracy by 10% on the mean error. Maximal errors in the range of 88% were encountered with rigid body component models and were reduced to 17% with flexible bodies. Finally, it was found that the hub rotating speed must be controlled to prevent unwanted gyroscopic efforts transmitted to the suspension. Since WFTs usually include transducers, wheel speed is readily available, and hub speed can therefore be controlled accordingly to improve frame load prediction accuracy.

In the current study, validity of the semi-analytical model was evaluated based on loads transmitted to the frame, obtained from a full analytical simulation. In fact, field tests should ideally include the following instrumentation: a steering wheel angle sensor, suspension displacements sensors for simulation validation purposes and an IMU to acquire trajectory data at a known vehicle location relative to the frame CoG. Subsequent work showed that IMUs often provide better speed signals than absolute position signals, so the CCRP method needs to be adapted to speed control instead of position control, with no alteration in the load prediction accuracy. The efforts applied by the CCRP method control algorithm compensate for the numerical errors that appear in the simulation, but also for the errors in the model; indeed, in an experimental situation, the non-suspended weight and CoG of the real vehicle might be different from those calculated in the simulation, for instance, due to the movements of the pilot’s body. This can lead to high correction efforts, but unless the vehicle frame is modeled as a flexible body, this has no impact on the mounting point loads.

Preliminary investigation of the model results in the frequency domain shows that the CCRP method is the best for implementing semi-analytical simulations. However, given that the maneuvers that were investigated had a limited frequency content, further investigation would be required for more diverse driving situations. The CCRP method introduced new natural frequencies owing to the PD controller connecting the frame to the marker that tracks the vehicle time trajectory. The control parameters must nonetheless be determined carefully to avoid generating errors in load predictions.

This study also highlighted limitations of the error map method, especially when phase offsets were present. Although a slight phase offset created high errors on the error maps, it has only a small impact on vehicle design and predicted fatigue life.
